# Association between urinary incontinence and sarcopenic obesity among middle-aged and older Brazilian women

**DOI:** 10.7717/peerj.20470

**Published:** 2026-01-14

**Authors:** Caroline Nayane Alves Medeiros, Ingrid Guerra Azevedo, Sabrina Gabrielle Gomes Fernandes, Álvaro Campos Cavalcanti Maciel, Saionara M.A. Câmara

**Affiliations:** 1 Physical Therapy Department—Post-Graduation Program of Physical Therapy, Universidade Federal do Rio Grande do Norte, Natal, Rio Grande do Norte, Brazil; 2 Academic Vicerrectory, Universidad Católica de Temuco, Temuco, La Araucanía, Chile

**Keywords:** Obesity, Sarcopenia, Urinary incontinence, Women

## Abstract

**Background:**

Urinary incontinence (UI) is a common complaint among middle-aged and older women, associated with many negative impacts in health. The aim of this study was to analyze whether an association exists between UI and sarcopenic obesity (SO) among middle-aged and older women living in northeastern Brazil. The association of UI with sarcopenia or obesity alone was also assessed.

**Methods:**

In a cross-sectional, analytical study, 531 women living in Santa Cruz and Parnamirim, Rio Grande do Norte state (Brazil), aged between 40 and 80 years, were assessed for the presence of UI in the last 12 months by self-reporting. Waist circumference equal to or greater than 88 cm was considered to classify obesity, while skeletal muscle mass below 5.93 kg/m^2^, assessed by bioelectrical impedance, classified the presence of sarcopenia. Based on these measurements, the participants were classified into four profiles: neither condition, sarcopenia, obesity, and sarcopenic obesity. Binary logistic regression investigated the association of UI with sarcopenia, obesity, and SO, adjusted for the covariates age, schooling, family income, marital status, hypertension, diabetes, parity, and menopausal status, considering *p* < 0.05.

**Results:**

A total of 10.7% had no sarcopenia or obesity, 10.7% had only sarcopenia, 69.7% had only obesity and 8.9% had SO. Obesity alone was associated with a higher odss of UI compared to the group with neither conditions (OR = 1.95; *p* = 0.025). The associations between UI and sarcopenia alone or combined with obesity were not significant.

**Conclusion:**

Obesity alone was associated with UI. The results highlight the need for screening UI symptoms among women with abdominal obesity for early detection and timely interventions.

## Introduction

Urinary incontinence (UI) is defined as the complaint of involuntary urine leakage during storage (D’ancona, Haylen & Abranches-Monteiro, 2019). Numerous negative impacts are associated with the presence of UI, including emotional aspects and reduced quality of life (Kwak, Kwon & Kim, 2016), depression (Lee, Rhee & Choi, 2021), social isolation (Yip et al., 2013), restrictions on social participation (Takahashi et al., 2015) and level of physical activity (Nygaard et al., 2005).

Among older women, abdominal obesity is one of the most clearly established risk factors for UI (Batmani et al., 2021). The increase in abdominal fat that generally accompanies female aging contributes to an increase in the demand for pelvic floor support, overloading these muscles (Suskind et al., 2017) and leading to the onset of UI symptoms. Sarcopenia, the reduction in muscle mass during the aging process, is another aspect that has been investigated in relation to UI (Erdogan et al., 2019). It is believed that sarcopenia may also affect the pelvic floor muscles, contributing to the higher incidence of UI among older individuals (Parker-Autry et al., 2017).

In this context, sarcopenia and obesity can occur concomitantly, leading to a condition called sarcopenic obesity (SO) (De Campos, Lourenço & Molina, 2021). The coexistence of obesity and sarcopenia appears to negatively impact muscle contraction quality, likely due to reduced protein synthesis caused by lipid infiltration into muscle tissue (Larsson et al., 2019). In fact, deterioration in lower-limb muscle performance has previously been reported for people with SO, as compared to people with only obesity or sarcopenia (Moreira et al., 2016). Since sarcopenia and obesity have been independently associated with UI previously (Chen, Jiang & Yao, 2023; Erdogan et al., 2019), we expected that individuals with both conditions would present an even higher probability of UI. Nevertheless, as far as we know, the synergistic effect of sarcopenia and obesity on UI has not been the focus of previous research.

Understanding how these conditions are associated with UI can help to understand the predisposing factors of this condition. Additionally, it can help to identify those with a greater probability of UI, towards whom prevention strategies should be directed.

In view of the above, the aim of this study is to analyze whether there is an association between SO and UI in middle-aged and older Brazilian women living in the northeast region of Brazil. The association of UI with sarcopenia or obesity alone was also assessed.

## Materials & Methods

### Type and study location

This was a cross-sectional study carried out in the cities of Parnamirim and Santa Cruz, in the state of Rio Grande do Norte, northeastern Brazil. The data were collected between 2014 and 2016, from the first follow-up of a longitudinal study that assessed hormone levels and their impact on sarcopenia and physical function in middle-aged and older women (Fernandes et al., 2020).

### Participants

A convenience sample was obtained after the project was publicized through advertisements in primary healthcare units and community centers in both cities. The sample included community-dwelling women aged between 40 and 80 years. Exclusion criteria were: the presence of neurological diseases, such as Parkinson’s disease, stroke, or any other condition that compromised the assessment of physical function measures, women who had undergone bilateral oophorectomy, since the original project included the assessment of hormone levels, and women with cognitive impairment identified by four errors or more in the Leganés Cognitive Test, a validated screening tool used to identify cognitive impairment (Caldas et al., 2012), which is considered indicative of the inability to complete the study procedures (Fernandes et al., 2020). Of the 589 participants assessed, five were excluded for being unable to self-report UI, and 53 because their records included insufficient data on the variables used to classify SO. The final sample of this study was 531 participants.

### Ethical aspects

All participants signed an informed consent form, and the study was approved by the Research Ethics Committee of the Federal University of Rio Grande do Norte (under No. 1.875.802).

### Procedures

All women were assessed by previously trained researchers at a community center in Parnamirim and at the the Federal University of Rio Grande do Norte campus, located in Santa Cruz. Standardized assessment protocols were applied, as described below.

#### Dependent variable: urinary incontinence

The participants were asked about the occurrence of UI in the last 12 months, based on the question “In the last 12 months, have you lost a small amount of urine, whether through straining, sneezing, coughing or accidentally?”. The possible answers were: once a week or more or every day, once a month or more, less than once a month, or none at all. Those who reported UI at any frequency were classified in one group as “yes”, while those who reported none we placed in another group (Markland et al., 2011).

#### Independent variable: sarcopenic obesity

For this sample, SO was defined based on the classification of the simultaneous presence of sarcopenia and obesity. To identify sarcopenia, appendicular skeletal muscle mass was assessed using the InBody R20 bioimpedance device (InBody 3.0, Biospace Co., Seoul, Korea). The device has eight electrodes, two for each extremity of the upper and lower limbs. A current of 250 mA, at frequencies of 20 and 100 kHz, was applied and the body composition is calculated according to the predictive equations provided by the equipment. The skeletal muscle mass index (SMMI) was then calculated from the sum of appendicular skeletal muscle mass (kg) divided by height (m) squared (Kim, Jang & Lim, 2016). Given the lack of consensus on the cutoff for low muscle mass among Brazilian populations, we adopted the 20th percentile of the sample distribution for classifying sarcopenia (≤5.93 kg/m^2^) as commonly used in other studies on the subject (Pagotto & Silveira, 2014; Cruz-Jentoft et al., 2010; Moreira et al., 2016). Although previous cutoffs for low muscle mass have been described in the literature, they consider only older populations, which could misclassify middle-aged women from our sample. Sensitivity analyses were conducted using the European Working Group on Sarcopenia in Older People (EWGSOP2) criterion for low muscle mass to classify sarcopenia (<5.5 kg/m^2^), as presented in the Supplemental Information (Cruz-Jentoft et al., 2019; Chen et al., 2014; Studenski et al., 2014). To assess of obesity, participants’ waist circumference was measured with a tape measure at the midpoint between the iliac crests and the lower rib margin, at the end of normal expiration. Those with a waist circumference ≥ 88 cm were classified as obese (Grundy et al., 2001).

Based on the above measurements, the participants were classified into four groups: (1) those with neither condition; (2) those with only obesity; (3) those with only sarcopenia; (4) those with obesity and sarcopenia simultaneously.

Weight (kg) and height (m) were measured using a Wiso^®^ W903 anthropometric scale and a Welmy^®^ stadiometer, respectively, to calculate the body mass index (BMI). A BMI ≥30 kg/m^2^ was used to define global obesity (World Health Organization, 2023). This classification was applied in sensitivity analyses of the association between SO and UI considering global obesity.

#### Covariates

The following variables were considered as potential covariates of the association between UI and SO, based on the literature.

(a) Demographic data and socioeconomic variables

Participants self-reported their age, schooling (less than primary, between primary and secondary, above secondary), color/ethnicity (white; brown or black), income (≥ 3 minimum wages; <3 minimum wages), and marital status (married or in a stable union; single, divorced or widowed).

Schooling, color/ethnicity, age, female gender, and marital status were considered covariates because a previous study showed that females, older people, those with low education and income, those of white ethnicity and those who are married are more likely to develop UI (Ganz et al., 2023).

(b) Comorbidities

The participants were asked if they had ever been diagnosed by a doctor or nurse with hypertension or diabetes. These chronic conditions have been associated with obesity, sarcopenia and UI (Patel et al., 2016; Gao et al., 2021; Batmani et al., 2021). Evidence suggests that women with chronic conditions, including diabetes, hypertension, pulmonary diseases, arthritis, and intestinal disorders, face a significantly higher risk of developing UI. Moreover, the presence of multiple chronic conditions appears to amplify this risk, particularly when combined with obesity, indicating a cumulative effect on the urinary function (Scime et al., 2022).

(c) Parity

Participants were classified according to their number of births (0–2 or ≥3). Evidence in the literature shows an association between parity and UI (Hage-Fransen et al., 2021). Parity is an important risk factor for female UI in fertile and early postmenopausal ages, with stronger associations for stress and mixed types of incontinence (Larsudd-Kåverud et al., 2022).

(d) Menopausal status

Participants were asked about the date of their last menstrual period to classify them as postmenopausal or not. Those who reported amenorrhea for at least one year or a history of hysterectomy (uterus removal) were classified as postmenopausal. Previous research has shown that the hypoestrogenism during this period is associated with a higher risk of developing UI and obesity (Lamerton, Torquati & Brown, 2018).

### Statistical analysis

A descriptive analysis of the sample was carried out according to the presence of SO, with medians and 25 and 75 quartiles for the quantitative variables, and absolute and relative frequencies for the categorical variables. The Chi-squared test assessed the association between the groups according to the presence of obesity and sarcopenia (neither condition, obesity, sarcopenia, and SO) and UI. The Mann–Whitney and Kruskall Wallis tests analyzed the associations between the quantitative variables with UI and SO. ANOVA with Tukey’s *post hoc* test was used to compare means of waist circumference and BMI among the SO groups. Binary logistic regression investigated the association between SO and UI groups adjusted for all potential covariates (age, education, family income, marital status, hypertension, diabetes, parity, and menopausal status), considering *p* < 0.05. To assess the robustness of the logistic regression model, given the small number of participants in the SO group, we conducted a nonparametric bootstrap with 1,000 resamples. Bias-corrected confidence intervals were calculated to evaluate the stability of regression coefficients, while the Hosmer-Lemeshow test was used to assess the goodness of fit of the model. Sensitivity analysis was conducted considering the SO groups based on BMI and the same covariates, as well as considering the EWGPSOP2 criterion for low muscle mass in the classification of sarcopenia. Finally, to check the consistency of the results, we performed a logistic regression model including the variables of abdominal obesity and sarcopenia separately and considering an interaction term between them. All analyses were carried out using SPSS, version 20.0 (IBM Corp., Armonk, NY, USA).

## Results

Of the 531 women included in the study, 57 (10.7%) were classified as having only sarcopenia, 370 (69.7%) as having only obesity, 47 (8.9%) as having sarcopenic obesity, and 57 (10.7%) with neither condition. The median age of the sample was 52 years. Table 1 shows the association between SO and the study’s covariates. Participants with SO had a higher median age (65 years) than the other groups (50 to 53 years) (*p* < 0.001). A greater proportion of those with SO had less than primary schooling (74.5%) compared to obesity (45.15%), sarcopenia (45.6%), and neither condition (33.3%) groups (*p* = 0.002). Women with neither condition showed lower prevalence of hypertension and diabetes compared to the other groups, as well as a lower proportion of postmenopausal women.

**Table 1:** Sample characteristics according to the presence of obesity, sarcopenia or sarcopenic obesity (*N* = 531).

**Variables**	**Neither condition**	**Sarcopenia**	**Obesity**	**Sarcopenic obesity**	*p*-value
	*n* = 57	*n* = 57	*n* = 370	*n* = 47	
	**n (%) or Median (q25; q75)**				
**Age**	50.0 (46.0; 54.0)	53.0 (50.0; 62.0)	52.0 (49.0; 58.0)	64.0 (55.0; 72.0)	<0.001
Color/Ethnicity					0.240^b^
White	18 (31.6%)	27 (47.4%)	129 (34.9%)	15 (31.9%)	
Black/Brown	39 (68.4%)	30 (52.6%)	241 (65.1%)	32 (68.1%)	
**Schooling**					0.002^b^
Less than primary school	19 (33.3%)	26 (45.6%)	167 (45.1%)	35 (74.5%)	
Between primary and secondary	25 (43.9%)	20 (35.1%)	147 (39.7%)	9 (19.1%)	
Above secondary	13 (22.8%)	11 (19.3%)	56 (15.1%)	3 (6.4%)	
**Income**					0.304^b^
Less than 3 MW	42 (73.7%)	38 (66.7%)	267 (72.2%)	39 (83.0%)	
Greater than or equal to 3 MW	15 (26.3%)	19 (33.3%)	103 (27.8%)	8 (17.0%)	
**Stable union**					0.883^b^
No	16 (28.1%)	20 (35.1%)	116 (31.4%)	15 (31.9%)	
Yes	41 (71.9%)	37 (64.9%)	254 (68.6%)	32 (68.1%)	
**Hypertension**					<0.001
No	48 (84.2%)	38 (66.7%)	191 (51.6%)	23 (48.9%)	
Yes	9 (15.8%)	19 (33.3%)	179 (48.4%)	24 (51.1%)	
**Diabetes**					0.035^b^
No	55 (96.5%)	51 (89.5%)	307 (83.0%)	38 (80.9%)	
Yes	2 (3.5%)	6 (10.5%)	63 (17.0%)	9 (19.1%)	
**Parity**					0.392^b^
0–2	27 (47.4%)	28 (49.1%)	146 (39.5%)	18 (38.3%)	
≥3	30 (52.6%)	29 (50.9%)	224 (60.5%)	29 (61.7%)	
**Postmenopausal**					0.032^b^
Yes	27 (47.4%)	40 (70.2%)	206 (55.7%)	32 (68.1%)	
No	30 (52.6%)	17 (29.8%)	164 (44.3%)	15 (31.9%)	

**Notes.**

q25: 25th quartile; q75: 75th quartile.

a *p*-value from the Kruskall Wallis test.

b *p*-value from the Chi-squared test.

The presence of UI was identified in 292 (55%) of the participants. Table 2 shows the associations between UI and the study variables. UI was more prevalent among women classified as obese (75%) compared to those without UI (63.2%) A statistically significant association was identified between UI and SO. A higher proportion of women with UI were classified as obese (75%) than the group without UI (63.2%) (*p* = 0.027). There was also a significant association in relation to parity, with a higher proportion of women with UI reporting three children or more than those without UI (63.0% *vs* 53.6%, respectively; *p* = 0.028). No statistically significant differences were observed between the groups in relation to other sociodemographic variables, comorbidities, or menopausal status. Similar results for the associations between SO and UI were observed when using the EWGSOP2 cutoff for low muscle mass to classify SO (Table S1).

**Table 2:** Associations between urinary incontinence and the study variables (*N* = 531).

**Variables**	**Urinary incontinence**		*p*-value
	No *n* = 239	Yes *n* = 292	
	**n (%) or Median (q25; q75)**		
**Sarcopenic obesity groups**			0.027^b^
Neither condition	33 (13.8%)	24 (8.2%)	
Sarcopenia	31 (13.0%)	26 (8.9%)	
Obesity	151 (63.2%)	219 (75.0%)	
Sarcopenic obesity	24 (10.0%)	23 (7.9%)	
**Age**	52.0 (49.0; 59.0)	53.0 (48.0; 60.0)	0.793^a^
Color/Ethnicity			0.142^b^
White	77 (32.2%)	112 (38.4%)	
Black/Brown	162 (67.8%)	180 (61.6%)	
**Schooling**			0.762^b^
Less than primary school	107 (44.8%)	140 (47.9%)	
Between primary and secondary	93 (38.9%)	108 (37.0%)	
Above secondary	39 (16.3%)	44 (15.1%)	
**Income**			0.592^b^
Less than 3 MW	171 (71.5%)	215 (73.6%)	
Greater than or equal to 3 MW	68 (28.5%)	77 (26.4%)	
**Stable union**			0.097^b^
No	84 (35.1%)	83 (28.4%)	
Yes	155 (64.9%)	209 (71.6%)	
**Hypertension**			0.293^b^
No	141 (59.0%)	159 (54.5%)	
Yes	98 (41.0%)	133 (45.5%)	
**Diabetes**			0.624^b^
No	205 (85.8%)	246 (84.2%)	
Yes	34 (14.2%)	46 (15.8%)	
**Parity**			0.028^b^
0-2	111 (46.4%)	108 (37.0%)	
≥3	128 (53.6%)	184 (63.0%)	
**Post-menopause**			0.511^b^
Yes	141 (59.0%)	164 (56.2%)	
No	98 (41.0%)	128 (43.8%)	

**Notes.**

q25: 25th quartile; q75: 75th quartile.

a *p*-value for the Mann–Whitney test.

b *p*-value for the Chi-square test.

MW Minimum wage

Table S2 shows the mean BMI and waist circumference across the SO groups. The group with SO had significantly lower BMI and waist circumference than the obesity-only group.

Table 3 shows the results of the binary logistic regression for UI in relation to the SO groups. The obesity-only group had a significantly higher odds of UI compared to the group with neither obesity nor sarcopenia, even after adjusting for potential covariates (OR = 1.92, 95% CI [1.07–3.45], *p* = 0.030). The sarcopenia-only and SO groups presented higher odds of UI, but the results failed to reach statistical significance (sarcopenia: OR = 1.13, 95% CI [0.53–2.47], *p* = 0.753; SO: OR = 1.21, 95% CI [0.52–2.84], *p* = 0.657). These results were confirmed by the linear regression model performed with sarcopenia and obesity as separate variables in the model, with an interaction term between them (Table S3). Table S4 shows the results of the binary logistic regression for UI in relation to SO, with obesity classification based on BMI. The obesity-only group continues to have a higher probability of presenting UI than the reference group (neither condition) (OR = 1.45, 95% CI [1.0–2.10], *p* = 0.05). Similar results were found when considering the EWGSOP2 cutoff of low muscle mass for the SO groups (OR = 1.77, 95% CI [1.08–2.90], *p* = 0.02) (Table S5).

**Table 3:** Binary logistic regression for urinary incontinence according to the presence of obesity, sarcopenia or sarcopenic obesity (*N* = 531).

**Sarcopenic obesity groups**	OR	CI 95%	*p*-value
Neither condition	1		
Sarcopenia	1.13	0.53; 2.47	0.753
Obesity	1.92	1.07; 3.45	0.030
Sarcopenic obesity	1.21	0.52; 2.84	0.657

**Notes.**

Model adjusted for age, race/ethnicity, schooling, family income, stable union, hypertension, diabetes, parity, and menopausal status.

OR Odds Ratio CI Confidence Interval

## Discussion

This study assessed the association between SO and UI in community-dwelling middle-aged and older women. The results show that women with obesity have a higher probability of presenting UI than those with neither obesity nor sarcopenia. The presence of sarcopenia alone or together with obesity was not associated with greater odds of UI.

Obesity is already well established as a risk factor for the development of UI (Chen, Jiang & Yao, 2023). It overloads the pelvic organs, ligaments, and pelvic floor muscles, as well as contributing to increased intra-abdominal and intravesical pressures (Swenson et al., 2017), favoring the occurrence of UI. Stress UI, the most common type, is characterized by involuntary urine leakage during physical effort and results from the urethra’s inability of to provide adequate resistance to urine flow under increased abdominal pressure (Jefferson & Linder, 2024). Obesity can also negatively influence the blood supply to the urethral sphincter muscles and increase urethral closure pressure values, promoting a pressure imbalance (Park & Lee, 2017). In addition, this condition can impair the quality of skeletal muscle contraction, through changes at cellular level, such as disruption of calcium signaling (Tallis, James & Seebacher, 2018).

Another common alteration resulting from obesity is the change in muscle fibers from slow to fast (Tallis, James & Seebacher, 2018). This leads to altered satellite cell function and has a negative impact on the formation of new muscles. It also contributes to changes in troponin isoforms responsible for cycling cross-bridges, an essential action for generating muscle contraction and consequent force production (Tallis, James & Seebacher, 2018). Thus, the contraction pattern of the pelvic floor skeletal muscles may deteriorate in obese people, predisposing them to UI. This is consistent with our findings, in which the prevalence of UI was higher among obese women than in the other groups.

In addition, the duration of overweight or obesity seems to influence the onset of UI during aging, as shown in a longitudinal cohort study of women aged between 50 and 79 years (Choi et al., 2022). These authors found that a longer duration of being overweight and obese significantly increased the probability of UI by 17% and 28%, respectively, compared to those without weight changes (Choi et al., 2022).

Abdominal obesity, in particular, seems to be a more important factor for UI than general obesity, as shown in a cohort study of 1,069 Brazilian women (≥60 years) (Krause et al., 2009). In this study, those with a waist circumference between 86 and 97 cm were 2.07 times more likely to have UI compared with women with lower measurements (Krause et al., 2009). This differs from the results found for obesity defined by BMI, which showed no statistically significant association with UI (Krause et al., 2009).

In the present study, sarcopenia was identified by the low level of appendicular muscle mass, and this may have a low correlation with the quantity and quality of pelvic floor muscles (PFM), which justifies the lack of association between the presence of sarcopenia and UI. In fact, a study evaluating the relationship between sarcopenia and PFM dysfunction in a sample of 217 Brazilian women (≥60 years) found no association between a probable diagnosis of sarcopenia (low handgrip strength) and UI or fecal incontinence, although an association was observed with pelvic organ prolapse (Silva et al., 2021). It should be noted that the authors classified sarcopenia based on appendicular muscle mass adjusted for participants’ weight, rather than for their height, as in the present study. It has been reported that adjusting muscle mass for body weight may result in misclassification of individuals who truly have sarcopenia (Tessier et al., 2019). Future studies investigating the relationship between the different ways of assessing sarcopenia and its association with UI may be useful for better understanding the discrepancies between the results.

It is also important to note that the participants in our sample who were classified as having sarcopenia had, on average, a lower BMI and waist circumference than the participants in the other groups (Table S1). Thus, although they may have worse muscle quality, their lower body weight may explain the lack of association between sarcopenia and UI.

However, contrary to our expectations, women with SO did not present a higher probability of UI, despite the presence of two factors expected to increase this likelihood. It is also important to note that participants with SO had lower mean BMI (27.0 *vs* 30.86, *p* < 0.001) and waist circumference (95.55 *vs* 100.28 *p* < 0.001), compared with those with obesity only (Table S1). In other words, they were less overweight or obese than the other group, which may help explain the lack of association between SO and UI. It is likely that the higher waist circumference observed in women with obesity alone is a factor that predisposes them more strongly to UI when compared with those with SO. Furthermore, it is possible that the cutoff used to define abdominal obesity in this study could not identify those with a greater probability of UI. Future studies are needed to determine which cutoff point can best identify individuals at highest risk of UI, in order to define the profile with the greatest incidence of this condition. The results of this study highlight the need to increase attention to the population of women with abdominal obesity, who are more likely to suffer from UI. Health professionals need to establish an early diagnosis of both obesity and UI in order to adopt effective strategies for appropriate treatment. These include reducing calorie intake (Schroder et al., 2021), physical exercise programs, and pelvic floor muscle training (Dumoulin et al., 2020). Such interventions can help mitigate the negative impact of UI on emotional well-being (Dasdelen et al., 2023), functional capacity, sexual function, and quality of life in this population (Porto et al., 2023).

This study has some limitations that need to be considered. There was a discrepancy in the number of participants across anthropometric profile groups, with smaller sample sizes in the sarcopenia and SO groups, which may have indirectly reduced the study’s statistical power. Although the results for the sarcopenia and SO groups indicated higher odds of UI compared with those with neither condition, they did not reach statistical significance. The wide 95% confidence intervals for these results may reflect the limited power of the analysis. Additionally, as highlighted previously, the SO group had lower BMI and waist circumference than the group with obesity only, which may have influenced the associations under study. Future studies may consider other measures to classify SO. In addition, the lack of an observed association between sarcopenia and UI in this study may also have been influenced by the method used to assess muscle mass. Bioelectrical impedance analysis was used to assess appendicular lean mass, which is an instrument for indirectly measuring muscle mass. Although it is practical for epidemiological studies, it may be less precise than other measures, such as the dual-energy X-ray absorptiometry (DXA). This potentially affected the identification of participants with sarcopenia and SO and, consequently, the detection of any true association with UI. Although this is not the gold standard method to assess muscle quantity, it is widely used in epidemiological research and indicated as a reliable alternative (Cruz-Jentoft et al., 2019). It is portable, low-cost, and does not expose subjects to radiation. Ultrasound-guided sarcopenia diagnosis has similar advantages and has been cited as a promising method in the diagnostic of sarcopenia (Staempfli et al., 2024). Future research may consider this method as a potential way to identify SO. As it was not possible to assess the specific muscle quantity of the PFM using electrical bioimpedance, it is suggested that further studies be carried out including specific methods for functional assessment of the PFM in particular, either through digital vaginal palpation (González-Castro et al., 2023) or perineometry (Bag Soytas et al., 2021). This allows the assessment of specific characteristics of these muscles among each anthropometric profile group. Furthermore, UI was assessed through a single self-reported question. This may introduce response bias, as participants may underreport or misclassify symptoms due to embarrassment or recall limitations. Furthermore, as this is a cross-sectional study, the associations observed do not allow for establishing causality. Despite this, these findings suggest the need for interventions, including dietary modifications and pelvic floor muscle training, mainly for women with obesity. Moreover, this study contributes to expanding evidence in a still underexplored field, namely the association between SO and UI among Brazilian women, reinforcing the need for public health strategies that address both sarcopenia and obesity in the prevention of UI.

## Conclusions

This study found an association between obesity and greater odds of UI in middle-aged and older women. This reinforces the importance of assessing waist circumference in clinical practice to identify individuals with abdominal obesity. Since UI is often underreported among women and has negative repercussions on emotional well-being, social participation, economic aspects, and quality of life, screening for UI in individuals with obesity allows for early detection and timely interventions, such as weight management, physical exercise, pelvic floor muscle training, and guidance on healthy lifestyle habits. Although SO was not associated with UI in this study, monitoring muscle function and body composition remains relevant for the prevention of age-related disorders. Further research is needed to assess the associations between SO and UI using different classification criteria, and how the simultaneous occurrence of these conditions can affect women’s health during aging.

##  Supplemental Information

10.7717/peerj.20470/supp-1 Supplemental Information 1 Raw data

10.7717/peerj.20470/supp-2 Supplemental Information 2 STROBE Checklist

10.7717/peerj.20470/supp-3 Supplemental Information 3 Codebook

10.7717/peerj.20470/supp-4 Supplemental Information 4 TCLE non blinded

10.7717/peerj.20470/supp-5 Supplemental Information 5 Association between urinary incontinence and sarcopenic obesity considering the EWGSOP2 cutoff on low muscle mass to classify sarcopenia A higher proportion of women with UI was classified as obese compared to the group without UI, considering the EWGSOP2 cutoff

10.7717/peerj.20470/supp-6 Supplemental Information 6 Averages of Body Mass Index and Waist Circumference among the anthropometric profiles (N=531) The mean BMI and waist circumference across the SO groups. The group with SO had significantly lower BMI and waist circumference than the obesity-only group.

10.7717/peerj.20470/supp-7 Supplemental Information 7 Binary logistic regression for urinary incontinence according to sarcopenia, obesity and interaction between sarcopenia and obesity (N= 531) The sarcopenia-only and obesity-only presented higher odds of UI, but the results failed to reach statistical significance for the interaction between sarcopenia and obesity.

10.7717/peerj.20470/supp-8 Supplemental Information 8 Supplementary Table 4- Binary logistic regression for urinary incontinence according to the anthropometric profiles considering Body Mass Index for obesity classification (N= 531) The obesity-only group has a higher probability of presenting UI when compared to the reference group (neither condition).

10.7717/peerj.20470/supp-9 Supplemental Information 9 Binary logistic regression for urinary incontinence according to sarcopenic obesity using the EWGSOP2 criterion of low muscle mass to classify sarcopenia (N= 531) The obesity-only group continues to have a higher probability of presenting UI when compared to the reference group (neither condition).

## Additional Information and Declarations

### Data Availability

The following information was supplied regarding data availability: The raw data is available in the Supplemental Files.
