# Supplementary material for: Association between urinary incontinence and sarcopenic obesity among middle-aged and older Brazilian women
Source: PeerJ. 2026 Jan 14;14:e20470. doi: 10.7717/peerj.20470 (PMC12811962; doi:10.7717/peerj.20470)
Supplement: Supplemental Information 3 [file peerj-14-20470-s003.docx]

**Supplemental File.** Codebook for SPSS data raw.

| *Variable and category* | *Values* |
| --- | --- |
| Age | Years |
| Color/Ethnicity |  |
| White | 1 |
| Black/Brown | 2 |
| Schooling |  |
| Less than primary | 0 |
| Between primary and secondary | 1 |
| Above secondary | 2 |
| Family_income |  |
| < 3 minimum wages (MW) | 0 |
| Greater than or equal to 3 minimum wages (MW) | 1 |
| Stable_union |  |
| No | 0 |
| Yes | 1 |
| Hypertension |  |
| No | 0 |
| Yes | 1 |
| Diabetes |  |
| No | 0 |
| Yes | 1 |
| Parity |  |
| 0-2 births | 0 |
| 3 or more births | 1 |
| Postmenopausal |  |
| No | 0 |
| Yes | 1 |
| Sarcopenic_obesity_categories |  |
| Normal | 1 |
| Sarcopenia | 2 |
| Obesity | 3 |
| Sarcopenic obesity | 4 |
| Urinary_incontinence |  |
| No | 0 |
| Yes | 1 |
| Waist | cm |
| Body Index Mass (BMI) | kg/m² |
| Sarcopenic_obesity_BMI |  |
| Normal | 1 |
| Sarcopenia | 2 |
| Obesity | 3 |
| Sarcopenic obesity | 4 |
