# Supplementary material for: Association between urinary incontinence and sarcopenic obesity among middle-aged and older Brazilian women
Source: PeerJ. 2026 Jan 14;14:e20470. doi: 10.7717/peerj.20470 (PMC12811962; doi:10.7717/peerj.20470)
Supplement: Supplemental Information 4 [file peerj-14-20470-s004.pdf]

UNIVERSIDADE FEDERAL DO RIO GRANDE DO NORTE
CENTRO DE CIÊNCIAS DA SAÚDE
PROGRAMA DE PÓS-GRADUAÇÃO EM FISIOTERAPIA

TERMO DE CONSENTIMENTO LIVRE E ESCLARECIDO – TCLE

Esclarecimentos

Este é um convite para você participar da pesquisa: INFLUÊNCIA DO STATUS MENOPAUSAL E NÍVEIS HORMONAIS NA FUNCIONALIDADE, DESEMPENHO MUSCULAR E COMPOSIÇÃO CORPÓREA: UM ESTUDO LONGITUDINAL, que tem como pesquisador responsável o prof.Dr. Álvaro Campos Cavalcanti Maciel da Universidade Federal do Rio Grande do Norte.

Esta pesquisa pretende investigar as alterações musculares como força, desempenho em atividades cotidianas e alterações na massa muscular corporal em mulheres em período menopausal ou após a menopausa, bem como a influência dos hormônios sexuais nessas possíveis alterações.

O motivo que nos leva a fazer este estudo é a necessidade de entender o processo de envelhecimento e suas consequências para auxiliar a elaboração de estratégias de prevenção e tratamento de incapacidade física.

Caso você decida participar, você deverá responder a uma entrevista para preenchimento de um formulário (chamado protocolo de pesquisa) para junção de dados sobre seu histórico ginecológico e menstrual; realizar uma avaliação física com: antropometria (medidas de circunferência abdominal e quadril, estatura e peso corporal), mensuração de pressão arterial e força muscular do membro superior e inferior dominantes, avaliação do desempenho funcional, função sexual e qualidade de vida, composição corporal (porcentagem de gordura corporal e massa muscular); e avaliação clínica (com médico ginecologista), além da realização de exames de sangue para avaliação de parâmetros bioquímicos e dosagem dos hormônios sexuais. Todos os procedimentos deverão ser realizados em 2 ou 3 dias diferentes, sendo necessária a disponibilidade de pelo menos 2 horas por dia. A pesquisa será realizada durante o período de 3 anos, com a realização de avaliações uma vez por ano, seguindo sempre o protocolo acima descrito.

Durante a realização do protocolo de pesquisa a previsão de riscos é mínima, ou seja, o risco que você corre é semelhante àquele sentido num exame físico de rotina. Pode acontecer um desconforto durante a coleta de sangue que será minimizado com compressas frias no local da picada da agulha, você terá como benefício principal a avaliação de seu estado de saúde. Ainda assim, na ocorrência de qualquer prejuízo comprovadamente decorrente desta pesquisa, a senhora será indenizada pelos pesquisadores responsáveis.

A sua primeira avaliação depende da manifestação do seu desejo em participar e agendamento na unidade do NIPEC/UNP (Rua Aspirante Santos,S/N – Santos Reis), no entanto, solicitamos que mantenha seus dados de telefone e endereço atualizados, pois nos anos seguintes deverá ser contatada por telefone ou convite no seu endereço.

Você tem o direito de se recusar a responder as perguntas, sem prejuízo a sua participação. Também pode recusar-se a participar em qualquer fase do estudo, a qualquer tempo, sem que seja causado constrangimento de qualquer natureza.

Em caso de algum problema que você possa ter, relacionado com a pesquisa, você terá direito a assistência que será prestada pelos pesquisadores preferencialmente através de recursos da rede pública de saúde e no impedimento e/ou limitação desta, pela rede privada sob custeio dos pesquisadores responsáveis.

Nenhuma terapia específica será aplicada, porém caso sejam identificadas alterações clínicas, patologias ou comprometimento à saúde que não tenham sido causadas pelo estudo, você será encaminhada para atendimento no serviço de saúde de referência dentro deste Município.

Durante todo o período da pesquisa você poderá tirar suas dúvidas ligando para Dr. Álvaro Campos Cavalcanti Maciel pelos telefones (84) 3342-2001; (84) 9129-6796, ou ainda, na Universidade Federal do Rio Grande do Norte no endereço: Campus Universitário, CP 1666 – Natal / RN, CEP: 59078 – 970.

Você tem o direito de se recusar a participar ou retirar seu consentimento, em qualquer fase da pesquisa, sem nenhum prejuízo para você.

Os dados que você irá nos fornecer serão confidenciais, sendo assegurada a sua privacidade conforme a Resolução nº 196/96 do Conselho Nacional de Saúde. Eles serão divulgados apenas em congressos ou publicações científicas, não havendo divulgação de nenhum dado que possa lhe identificar.

Esses dados serão guardados pelo pesquisador responsável por essa pesquisa em local seguro e por um período de 5 anos.

Se você tiver algum gasto pela sua participação nessa pesquisa, ele será assumido pelo pesquisador e reembolsado para você.

Qualquer dúvida sobre a ética dessa pesquisa você deverá ligar para o Comitê de Ética em Pesquisa da Universidade Federal do Rio Grande do Norte, telefone 3215-3135.

Este documento foi impresso em duas vias. Uma ficará com você e a outra com o pesquisador responsável: Dr. Álvaro Campos Cavalcanti Maciel.

Consentimento Livre e Esclarecido

Após ter sido esclarecido sobre os objetivos, importância e o modo como os dados serão coletados nessa pesquisa, além de conhecer os riscos, desconfortos e benefícios que ela trará para mim e ter ficado ciente de todos os meus direitos, concordo em participar da pesquisa INFLUÊNCIA DO STATUS MENOPAUSAL E NÍVEIS HORMONAIS NA FUNCIONALIDADE, DESEMPENHO MUSCULAR E COMPOSIÇÃO CORPÓREA: UM ESTUDO LONGITUDINAL, e autorizo a divulgação das informações por mim fornecidas em congressos e/ou publicações científicas desde que nenhum dado possa me identificar.

Nome:____

Local: _________________ Data: _____/______/_______

Assinatura_____________________________________

Declaração do pesquisador responsável

Como pesquisador responsável pelo estudo INFLUÊNCIA DO STATUS MENOPAUSAL E NÍVEIS HORMONAIS NA FUNCIONALIDADE, DESEMPENHO MUSCULAR E COMPOSIÇÃO CORPÓREA: UM ESTUDO LONGITUDINAL, declaro que assumo a inteira responsabilidade de cumprir fielmente os procedimentos metodologicamente e direitos que foram esclarecidos e assegurados ao participante desse estudo, assim como manter sigilo e confidencialidade sobre a identidade do mesmo.

Declaro ainda estar ciente que na inobservância do compromisso ora assumido estarei infringindo as normas e diretrizes propostas pela Resolução 466/12 do Conselho Nacional de Saúde – CNS, que regulamenta as pesquisas envolvendo o ser humano.

___________________, ________de _______________de _______.

____

Assinatura do pesquisador responsável
