# Supplementary material for: Association between urinary incontinence and sarcopenic obesity among middle-aged and older Brazilian women
Source: PeerJ. 2026 Jan 14;14:e20470. doi: 10.7717/peerj.20470 (PMC12811962; doi:10.7717/peerj.20470)
Supplement: Supplemental Information 5 — A higher proportion of women with UI was classified as obese compared to the group without UI, considering the EWGSOP2 cutoff [file peerj-14-20470-s005.docx]

**Supplementary Material**

Supplementary table 1: Association between urinary incontinence and sarcopenic obesity considering the EWGSOP2 cutoff on low muscle mass to classify sarcopenia.

|  | **Urinary incontinence** | |  |
| --- | --- | --- | --- |
|  | No  n= 239 | Yes  n= 292 | p value |
|  | N (%) | |  |
| **Sarcopenic Obesity** |  |  | 0.012 |
| Normal | 47 (19.7%) | 37 (12.7%) |  |
| Sarcopenia | 17 (7.1%) | 13 (4.5%) |  |
| Obesity | 164 (68.6%) | 236 (80.8%) |  |
| Sarcopenic Obesity | 11 (4.6%) | 6 (2.1%) |  |

CI: Confidence Interval, EWGSOP2: European Working Group on Sarcopenia in Older People (2018), OR: Odds Ratio.
