# Supplementary material for: Association between urinary incontinence and sarcopenic obesity among middle-aged and older Brazilian women
Source: PeerJ. 2026 Jan 14;14:e20470. doi: 10.7717/peerj.20470 (PMC12811962; doi:10.7717/peerj.20470)
Supplement: Supplemental Information 6 — The mean BMI and waist circumference across the SO groups. The group with SO had significantly lower BMI and waist circumference than the obesity-only group. [file peerj-14-20470-s006.docx]

**Supplementary Material**

**Supplementary Table 2-** Averages of Body Mass Index and Waist Circumference among the anthropometric profiles (N=531).

|  | **Neither condition** | **Sarcopenia** | **Obesity** | **Sarcopenic Obesity** | ***p*-value** |
| --- | --- | --- | --- | --- | --- |
| **BMI (kg/m²)** | 25.16 (±2.35) | 22.77 (±2.21) | 30.86 (±4.16) | 27.00 (±2.72) | <0.001^a^ |
| **WC (cm)** | 83.03 (±4.61) | 81.01 (±5.22) | 100.28 (±8.37) | 95.55 (±5.08) | <0.001^b^ |

^a^: Significant difference between all groups except between Normal with Sarcopenia, and with Sarcopenic Obesity. ^b^: significant difference between all groups except Normal and Sarcopenia. BMI: Body Mass Index. WC: Waist circumference. Kg/m²: kilogram per square meter.
