# Supplementary material for: Association between urinary incontinence and sarcopenic obesity among middle-aged and older Brazilian women
Source: PeerJ. 2026 Jan 14;14:e20470. doi: 10.7717/peerj.20470 (PMC12811962; doi:10.7717/peerj.20470)
Supplement: Supplemental Information 7 — The sarcopenia-only and obesity-only presented higher odds of UI, but the results failed to reach statistical significance for the interaction between sarcopenia and obesity. [file peerj-14-20470-s007.docx]

**Supplementary Material**

Supplementary table 3: Binary logistic regression for urinary incontinence according to sarcopenia, obesity and interaction between sarcopenia and obesity (N= 531).

| **Sarcopenic Obesity** | **OR** | **95% CI** | | **p** |
| --- | --- | --- | --- | --- |
| Sarcopenia (yes) | 1.13 | 0.53; | 2.43 | 0.75 |
| Obesity (yes) | 1.92 | 1.07; | 3.45 | 0.03 |
| Sarcopenia*Obesity | 0.56 | 0.21; | 1.50 | 0.25 |

Model adjusted for age, race/ethnicity, schooling, family income, stable union, hypertension, diabetes, parity and menopausal status. CI: Confidence Interval, OR: Odds Ratio.
