# Supplementary material for: Association between urinary incontinence and sarcopenic obesity among middle-aged and older Brazilian women
Source: PeerJ. 2026 Jan 14;14:e20470. doi: 10.7717/peerj.20470 (PMC12811962; doi:10.7717/peerj.20470)
Supplement: Supplemental Information 8 — The obesity-only group has a higher probability of presenting UI when compared to the reference group (neither condition). [file peerj-14-20470-s008.docx]

**Supplementary Material**

**Supplementary Table 4-** Binary logistic regression for urinary incontinence according to the anthropometric profiles considering Body Mass Index for obesity classification (N= 531).

| **Anthropometric profile** | **OR** | **CI 95%** | ***p*-value** |
| --- | --- | --- | --- |
| Neither condition | 1 |  |  |
| Sarcopenia | 1.43 | 0.30; 6.87 | 0.65 |
| Obesity | 1.45 | 1.00; 2.10 | 0.05 |
| Sarcopenic Obesity | 1.97 | 0.17; 22.72 | 0.32 |

Model adjusted for age, schooling, income, stable union, hypertension, diabetes, parity, and menopausal status. OR: Odds Ratio; CI: Confidence Interval.
