# Supplementary material for: Association between urinary incontinence and sarcopenic obesity among middle-aged and older Brazilian women
Source: PeerJ. 2026 Jan 14;14:e20470. doi: 10.7717/peerj.20470 (PMC12811962; doi:10.7717/peerj.20470)
Supplement: Supplemental Information 9 — The obesity-only group continues to have a higher probability of presenting UI when compared to the reference group (neither condition). [file peerj-14-20470-s009.docx]

**Suplementary Material**

**Supplementary table 5:** Binary logistic regression for urinary incontinence according to sarcopenic obesity using the EWGSOP2 criterion of low muscle mass to classify sarcopenia (N= 531).

| **Sarcopenic Obesity** | **OR** | **95% CI** | **p** |
| --- | --- | --- | --- |
| Normal | 1 |  |  |
| Sarcopenia | 0.95 | 0.40; 2.26 | 0.90 |
| Obesity | 1.77 | 1.08; 2.90 | 0.02 |
| Sarcopenic Obesity | 0.61 | 0.19; 1.95 | 0.41 |

Model adjusted for age, race/ethnicity, schooling, family income, stable union, hypertension, diabetes, parity and menopausal status. CI: Confidence Interval, EWGSOP2: European Working Group on Sarcopenia in Older People (2018), OR: Odds Ratio.
